# Some Aspects of Time-Reversal in Chemical Kinetics

**DOI:** 10.3390/e22121386

**Published:** 2020-12-07

**Authors:** Ulrich Maas

**Affiliations:** Institut für Technische Thermodynamik, Karlsruher Institut für Technologie, 76131 Karlsruhe, Germany; Ulrich.Maas@kit.edu

**Keywords:** chemical kinetics, time reversal, time scales, low-dimensional manifolds

## Abstract

Chemical kinetics govern the dynamics of chemical systems leading towards chemical equilibrium. There are several general properties of the dynamics of chemical reactions such as the existence of disparate time scales and the fact that most time scales are dissipative. This causes a transient relaxation to lower dimensional attracting manifolds in composition space. In this work, we discuss this behavior and investigate how a time reversal effects this behavior. For this, both macroscopic chemical systems as well as microscopic chemical systems (elementary reactions) are considered.

## 1. Introduction

**Figure 1:**
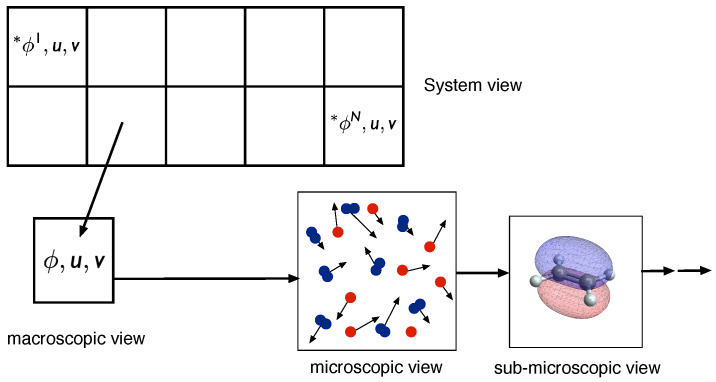
Different levels of chemical kinetics (see respective sections for notation): systems (I), macroscopic view (II), microscopic view (III), sub-microscopic view (IV).

The thermodynamic time arrow is based on the second law of thermodynamics: entropy increases with time. In thermodynamic equilibrium, however, no distinction between past and future can be made. In chemical systems, the dynamics towards chemical equilibrium is governed by chemical kinetics. The description of chemical kinetics is a difficult task because different hierarchical levels are involved (see Figure 1). In typical reacting flows, an overall system is described by the time- and space dependent fields of mean velocities of the different species involved, and the time- and space dependent thermokinetic states. For chemically reacting flows, in many cases, a continuum description can be used, which relies on macroscopic balance equations for mass, momentum, energy, and species masses. These equations have been discussed in detail in many articles [1,2,3].

It is remarkable that the constitutive relations (transport coefficients) in these balances can be recovered from the microscopic level (kinetic theory of gases) [1], and many theories exist, which relate the macroscopic chemical rate equation to microscopic processes such as collisions between molecules, formation of activated complexes, formation and braking of chemical bonds, etc. (see [4,5,6,7] for references).

A profound simplification is obtained if we restrict ourselves to pure chemical kinetic systems, which are assumed to be homogeneous, and therefore only time dependent. For this purpose, we shall treat reacting systems (cf. Figure 1) as an ensemble of *N* different homogeneous reactors, which do not interact (interactions due to heat and mass transfer themselves are interesting to be investigated, but add complexity to the analysis and are therefore not subject of the following analysis). Based on this view of chemically reacting systems, we can identify four different hierarchical levels (see Figure 1), namely

- System view (I): level at which the statistics of the overall reacting flow can be investigated
- Macroscopic view (II): level at which the chemical kinetics is governed by macroscopic variables such as mass fractions, specific internal energy, and specific volume (or concentrations, temperature, add pressure), and which is described by large sets of elementary reactions
- Microscopic view (III): level at which elementary reactions occur. This level is governed by collisions and interactions of different molecules, and depends on the translational, rotational, vibrational, and electronic states of the molecules
- Sub-microscopic view (IV): This level describes the energies of the quantum states (inlet figure produced using the Wolfram Demonstrations project [8])

Below this last hierarchical level, there are of course even smaller levels, governed by quantum-mechanical processes, which shall not be considered here. The hierarchy of the chemical kinetics simplifies the detailed investigation considerably because it allows a decoupling of the different levels (although it is known that in some chemically reacting systems such a decoupling can lead to large errors in the quantitative description, and, e.g., state selective reaction mechanisms have to be used for the description of macroscopic systems [9,10,11]).

As stated above, the arrow of time is based on the second law of thermodynamics. The dynamics of chemically reacting systems evolve with time until thermal, mechanical, and chemical equilibrium is attained. This natural direction of time brings up the question of what happens for a “time reversal”. Here, we have to distinguish between the question of specifying the state of a system at t−Δt (Δt>0) given the state of system at t=0 (deterministic view back in time), and the question of what would happen in case of a time reversal (leading to an entropy decrease with time). This is directly related to the question of causality. For causality, the reaction of say A+B→C depends on the presence of A and B, and entropy increases with increasing time, whereas, for anti-causality, it depends on the presence of C, and entropy decreases with time [12].

The questions thus can be summarized as:

- Time reversal and anti-causality: How do the kinetics behave if the arrow of time reverses? (This question will not be discussed in this work.)
  – Is a purely macroscopic view possible?
  – Does the “negative time” scale in a way different from the regular time?
  – Is there a similarity in the hierarchical nature of the chemical kinetics?
- Time reversal and causality: Let us assume that we know the thermokinetic state of a system at t=0. Then, can we determine the state that the system had at −Δt (with Δt>0)?
  – Is there a limiting value for t<0?
  – How is the hierarchical nature of the chemical kinetics reflected by a time reversal?
  – For t→∞, the chemical kinetics yield the thermodynamic equilibrium. What happens for t→−∞?

The answers to these questions are very challenging because there is a hierarchy involved in the chemical reaction ranging from the microscopic level (collisions between molecules, redistribution of the internal degrees of freedom, breaking and formation of bonds) up to the macroscopic level (description of a chemical reaction by a rate coefficient, which depends on temperature and in some cases on pressure, where the states of the reactants are assumed to be in thermal equilibrium [11]).

In the following, we shall address the questions arising for time reversal on the different hierarchical levels, starting with the macroscopic level. We shall only focus on aspects which are based on causality, and not discuss aspects concerning a change of the arrow of time, although such aspects have interesting implications in the kinetics of anti-matter [13,14].

## 2. The Macroscopic View

### 2.1. The Forward Behavior

Let us start with a brief discussion of the forward behavior of a chemical reaction system involving $n_{s}$ species. We shall focus on a homogeneous reaction system and allow several thermodynamic constraints (e.g., constant internal energy, or constant enthalpy, constant pressure, or constant volume. These constraints enter the system of governing equations as parameters ξ (note that the parameters could be themselves treated as variables of the system, leading to equations for the state space [15]), and we are left with the investigation of the $n_{s}$-dimensional composition space only. The composition vector shall be given by the vector of species specific mole numbers

(1) $$\phi ={\left(\phi_{1},\phi_{2},\ldots ,\phi_{n_{s}}\right)}^{T}$$

with the specific mole numbers defined as $\phi_{i}=w_{i}/M_{i}$, where $w_{i}$ is the mass fraction and $M_{i}$ the molar mass of species *i*, respectively (the formulation simplifies the notation for the element conservation constraints). Physical constraints like positiveness of mass fractions limit the allowed composition space Σ to a domain (note that positiveness of temperature could also be enforced, but we shall not make use of this constraint), where

(2) $$\Sigma =\left\{\phi |\phi_{i}\geq 0,i=1,\ldots ,n_{s}\wedge \sum_{j=1}^{n_{s}}M_{j}\phi_{j}=1\right\}.$$

We assume that this vector evolves according to the chemical rate equations. The complex reaction mechanism shall comprise $n_{r}^{+}$ elementary forward reactions, and each shall be accompanied by a corresponding reverse reaction, such that the equilibrium determined by the kinetic equations is consistent with the thermodynamic equilibrium [16]. Furthermore, we assume that the remaining conditions necessary for consistency with thermodynamics (e.g., the fact that all chemical elements in the system take place in chemical reactions, and the detailed balance is fulfilled [17,18]) are also met.

The chemical reaction *l* ($1\leq l\leq n_{r}=2n_{r}^{+}$) shall be given in the general form

(3) $${\hat{a}}_{1l}A_{1}+{\hat{a}}_{2l}A_{2}+\ldots +{\hat{a}}_{n_{s}l}A_{n_{s}}⟶{\tilde{a}}_{1l}A_{1}+{\tilde{a}}_{2l}A_{2}+\ldots +{\tilde{a}}_{n_{s}l}A_{n_{s}}$$

or written as sums

(4) $$\sum_{i=1}^{n_{s}}{\hat{a}}_{il}A_{i}⟶\sum_{i=1}^{n_{s}}{\tilde{a}}_{il}A_{i}$$

with the stoichiometric coefficients ${\hat{a}}_{il}$ and ${\tilde{a}}_{il}$ of the species *i* in reaction *l*, $A_{i}$ as the species symbols. Then, the molar chemical rate of production $\omega_{i}$ and the chemical production term $\Omega_{i}$ of species *i* is given as

(5) $$\Omega_{i}=\sum_{l=1}^{n_{r}}\left({\tilde{a}}_{il}-{\hat{a}}_{il}\right)\frac{r_{l}}{\rho }\;.$$

If $\Omega \left(\phi ,\xi \right)={\left(\Omega_{1},\Omega_{2},\ldots ,\Omega_{n_{s}}\right)}^{T}$ denotes the chemical source (or sink) term, *R*, the ($n_{s}\times n_{r}$)–dimensional matrix of stoichiometric coefficients ($R_{il}={\tilde{a}}_{il}-{\hat{a}}_{il}$), and *r* the $n_{cr}$–dimensional vector of reaction rates ($r={\left(r_{1},r_{2},\ldots ,r_{c}\right)}^{T}/\rho$), then the notation simplifies to

(6) Ω(ϕ;ξ)=Rr(ϕ;ξ).

Note that, due to element conservation, the row rank of *R* is equal to $n_{r}-n_{e}$, where $n_{e}$ is the number of different elements.

The vector ϕ evolves according to

(7) $$\frac{\partial \phi (t)}{\partial t}=\Omega \left(\phi \left(t\right);\xi \right)\;\;\phi \left(t=0\right)=\phi^{0}$$

For simplicity, we assume that the additional thermodynamic variables ξ (e.g., overall mass, specific volume, specific internal energy) are constant and do not involve an explicit time dependence. Therefore, these variables are mostly dropped in the following notation.

There are several things to note with respect to this equation:

- it is an $n_{s}$-dimensional system of stiff nonlinear ordinary differential equations
- it is an initial value problem
- the solution for t→∞ yields the chemical equilibrium $\phi^{e}=\phi^{e}\left(\xi ,\chi \right)$. This equilibrium value is a function of thermodynamic variables ξ and the elemental composition ($n_{e}$ elements), where χ denotes the vector of element mass fractions, which is a function of the specific mole numbers.
- assuming that the chemical rates are Lipschitz continuous, the Picard–Lindelöf theorem states that the initial value problem has a unique solution
- the system is “deterministic”
- the dynamics takes place in the so-called reaction space [19], which has a dimension of $n_{s}-n_{e}$ because the elements are conserved in chemical reaction

It can be shown (at least for ideal gases) that the final chemical equilibrium for t→∞ is in general unique and exists for different reaction conditions (isothermal and isobaric, adiabatic and constant specific volume, etc.). In [20], a detailed discussion and many references to previous work on this topic can be found. See also [21] for a discussion of the dynamics of chemical reaction systems towards stationary states.

Noting that the solution trajectory of Equation (7) is given as

(8) $$\phi \left(t\right)=\phi^{t}\left(t,\phi^{0};\xi \right)),$$

we can state an important property with respect to time: unless a state ϕ(t=0) corresponds to a stationary value (e.g., the equilibrium), the state $\phi \left(t<0\right)$ in the past is explicitly known (at least for times larger than a certain limit, see below). This means that, based on a current state, we can make statements on the past of this state.

**Figure 2:**
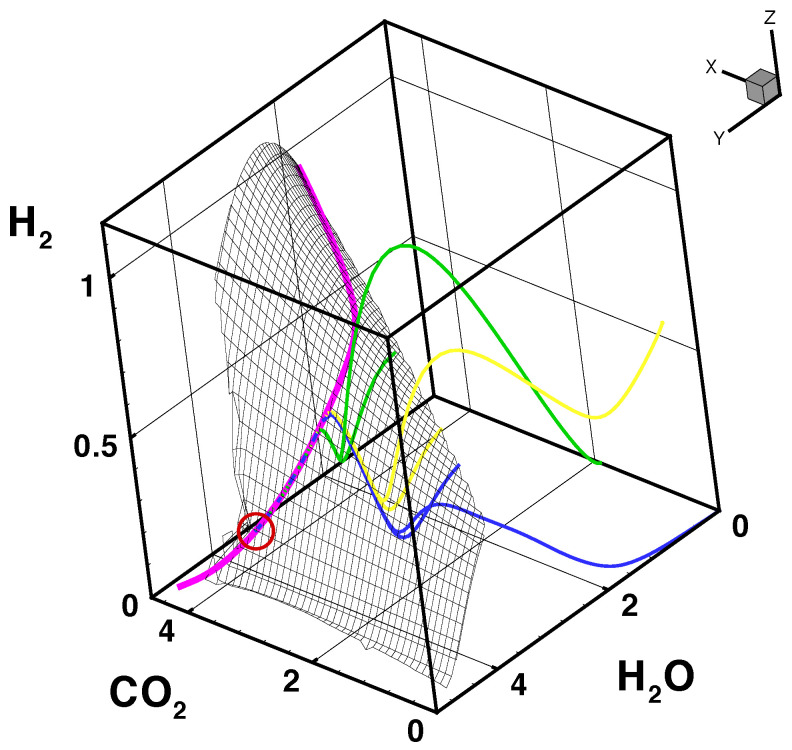
Results of three sample homogeneous reactor calculations ($i-C_{8}H_{18}$ (blue), ${\mathrm{CH}}_{4}+{\mathrm{CO}}_{2}$ (green), and $C_{2}H_{2}+{\mathrm{CH}}_{4}+H_{2}+{\mathrm{CO}}_{2}$ (yellow)) and the corresponding trajectories of reaction progress (corresponding lines on the surface) on a two-dimensional iso-octane/air ILDM (mesh). All the trajectories are attracted toward the two-dimensional ILDM (mesh), then toward the one-dimensional ILDM (magenta line), and finally end in the equilibrium value (circle), figure adopted from [22].

Some issues of the dynamics of chemical reaction systems can be illustrated using an example from combustion kinetics [22]. Figure 2 shows example solutions of trajectories for stoichiometric mixtures of different compounds. The initial mixtures have only in common that they have the same element composition χ, pressure, and specific enthalpy. Otherwise, they are quite different, namely one mixture is a stoichiometric mixture of iso-octane with air, the second is a mixture of methane and ${\mathrm{CO}}_{2}$ with air, and the third is a mixture of acetylene, methane, hydrogen, and ${\mathrm{CO}}_{2}$ with air. Chemical reaction corresponds to a movement along a trajectory in the $n_{s}$-dimensional composition space. The original composition space has quite a high dimension [22]. Therefore, plotted here is only a projection into the subspace of specific mole numbers of $H_{2}$, ${\mathrm{CO}}_{2}$, and $H_{2}O$. Several interesting properties of the dynamics of the system can be seen from Figure 2:

- The trajectories each start a given initial value
- The trajectories do not cross (an apparent crossing in the figure is only a result of the projection of the multi-dimensional composition space onto a three-dimensional space
- The trajectories evolve in time towards the equilibrium point
- There is a transient relaxation towards low-dimensional manifolds, it is observable in Figure 2 in the three-dimensional projection the final relaxation towards a two-dimensional manifold, then a one-dimensional manifold, and finally the zero-dimensional manifold (note that the trajectories are chosen such that they have the same equilibrium value).
- starting from very different initial conditions, the states get closer and closer, this means that the system seemingly loses information on its past.

The loss of information on the past is, however, only apparently there because, as stated above, we can recover the past from the present due to the deterministic nature of the macroscopic equation.

### 2.2. Intrinsic Low-Dimensional Manifolds (ILDM)

Simple models describe the chemical reaction according to the assumption that the chemical reaction is in thermodynamic equilibrium. It is assumed that the chemical equilibrium is attained infinitely fast, or at least very fast compared to the time scales of the physical processes such as mixing or flow. However, due to this simplification, all information on the dynamics of the chemical reaction is lost, and the fact that chemistry is only finitely fast (and thus possibly rate-determining) is completely neglected. On the other hand, detailed reaction mechanisms describe the full dynamics of the chemical reaction system. Even processes that are very fast and therefore not rate-determining are dealt with in an accurate way. It is well known, however, that the chemical kinetics have a hierarchical structure [22,23], caused by

- the existence of disparate time scales
- the fact that most time scales have a dissipative nature
- the existence of low-dimensional attractors in composition space (cf. also Figure 2)

The existence of low-dimensional manifolds allows for separating the slow and the fast dynamics of the chemical reactions by projecting the governing equation system (7) onto the (in general nonlinear) slow subspace. A variety of different methods are available to identify such low-dimensional manifold, such as: “Trajectory Generated Manifolds” [24], Repro-modeling [25,26], correlation of DNS data [27], slow manifolds [23,28,29,30], the method of invariant grids [31], minimal entropy production trajectories [32], CSP-Manifolds [30,33,34,35,36,37], Intrinsic Low-Dimensional Manifolds [15,38,39], TILDM [40] or GQL [41,42] techniques, and for systems coupled with molecular transport processes Flamelet Generated Manifolds [43], multi-dimensional flamelets [44], and Reaction–Diffusion Manifolds [45,46].

In the following, we shall limit our analysis to attracting low-dimensional manifolds based on ILDM [38] or GQL (“global quasi-linearization”) [41]. Note that for linear chemical systems these methods are equivalent.

The eigenvalues of the Jacobian $\Omega_{\phi }$ (${\left(\Omega_{\phi }\right)}_{i,j}=\partial \Omega_{i}/\partial \phi_{j}$ (or in the case of GQL the GQL-matrix [41]) identify the time-scales of the dynamic system and the eigenvectors the associated characteristic directions (see, e.g., [19]). The Jacobian (or the GQL-matrix) can be decomposed into a slow and a fast subspace according to

(9) $$\Omega_{\phi }=\left(Z_{s}Z_{f}\right)\left(\begin{array}{cc} N_{s} & 0\\ 0 & N_{f} \end{array}\right)\left(\begin{array}{c} {\hat{Z}}_{s}\\ {\hat{Z}}_{f} \end{array}\right),$$

where $Z_{s}$ is the $n_{s}$ by $m_{s}$-dimensional matrix which forms an invariant subspace associated with the $m_{s}$ eigenvalues having the largest real parts (these include the $n_{e}$ zero eigenvalues corresponding to element conservation), and $Z_{f}$ the $n_{s}$ by $m_{f}$-dimensional matrix which forms an invariant subspace associated with the $m_{f}=n_{s}-m_{s}$ eigenvalues having the smallest real parts (degenerate eigenvalues are counted multiple). $N_{s}$ is an $m_{s}$ by $m_{s}$, and $N_{f}$ an $m_{f}$ by $m_{f}$-dimensional matrix associated with the eigenvalues. ${\tilde{Z}}_{f}$ is the $m_{f}$ by $n_{s}$-dimensional matrix which forms a left invariant subspace and can be obtained via:

(10) $$\left(\begin{array}{c} {\hat{Z}}_{s}\\ {\hat{Z}}_{f} \end{array}\right)={\left(Z_{s}Z_{f}\right)}^{-1}.$$

The decomposition is performed such that $ℜ\left(\lambda_{i}\left(N_{s}\right)\right)\gg ℜ\left(\lambda_{i}\left(N_{f}\right)\right)\wedge ℜ\left(\lambda_{i}\left(N_{f}\right)\right)<0\forall i,j\in N(1\leq i\leq m_{s},1\leq j\leq m_{f})$ (*ℜ* denotes the real part; for a discussion of the splitting and the handling of complex or degenerate eigenvalues, see [15,19]). Note that these equations can also be re-formulated in terms of concentrations, which is done for the simple test case presented below.

For the chemical system (7) specified above, GQL and ILDM define the *m*-dimensional manifolds in composition space for a given element composition $\chi^{0}$ and given values $\xi^{0}$ of the thermodynamic variables (internal energy and specific volume, enthalpy and pressure, or temperature and pressure) low-dimensional manifolds as:

(11) $$M^{m}=\left\{\phi |{\tilde{Z}}_{f}\left(\phi ;\xi^{0}\right)\Omega \left(\phi \left(t\right);\xi^{0}\right)=0\wedge \chi \left(\phi \right)=\chi^{0}\right\}$$

This equation ${\tilde{Z}}_{f}$, which may depend on ϕ and ξ, denotes the left invariant subspace of the GQL-matrix or the Jacobian matrix (for ILDM). This means that the rates of the chemical processes in the direction of the fast eigensystem vanish. By solving the manifold equation, an explicit formulation for the low-dimensional manifold can be obtained in the form

(12) $$M^{m}=\left\{\phi \left(\theta \right)|\theta \in P\right\},$$

where P denotes the domain of parameters θ of the manifold. For the example shown in Figure 2, the number $m_{s}$ of slow processes is $n_{e}+1$ for the one-dimensional manifold and $n_{e}+2$ for the two-dimensional manifold (note that the analysis is performed for one given element composition). From Figure 2, it can be seen clearly that the dynamics of the kinetics is governed by a successive relaxation to lower and lower-dimensional manifolds. This is quite a general behavior of complex kinetic systems and observed not only for combustion mechanisms, but also for atmospheric chemistry [47] or in systems biology [48]. Although the concept of low-dimensional manifolds has been developed (and can be applied) for the general case of nonlinear source-terms (like in the example of Figure 2), we shall use for illustration purposes a linear test case.

#### A Simple Linear Test Case

Linear test cases have been used for a long time to analyze the dynamics of the kinetics due to their simplicity [17]. In order to analyze the dynamic behavior of chemical systems, we shall also use a simple linear test case comprising only first order reactions:

(13) $$A\underset{b}{\overset{a}{⇌}}B\underset{d}{\overset{c}{⇌}}C$$

leading to the ordinary differential equation system, which after non-dimensionalization reads:

(14) $$\begin{array}{cccc} \frac{dx}{dt} & = & -ax+by & \\ \frac{dy}{dt} & = & ax+by & -cy+dz\\ \frac{dz}{dt} & = & \; & cy-dz, \end{array}$$

where x,y,z denote the concentrations of $c_{A},c_{B},c_{C}$, respectively, and a,b,c, and *d* are the rate coefficients of the different first order reactions. Note that this is a simple linear system, which has been chosen to simplify the description. Nonlinear models involving bi-molecular or tri-molecular reactions can be handled in a similar way [19]. In vector notation with $u={\left(x,y,z\right)}^{T}$, the equation reads:

(15) $$\frac{du}{dt}=\underset{J}{\underset{︸}{\left(\begin{array}{ccc} -a & b & 0\\ a & -b-c & d\\ 0 & c & -d \end{array}\right)}}u=Ju\;u\left(t=0\right)=u_{0}={\left(x_{0},y_{0},z_{0}\right)}^{T},$$

and an analytical solution is given by:

(16) $$u=e^{Jt}u_{0}$$

The solution can be easily obtained using e.g., Mathematica [49] and is given in Appendix A for the case of a=1,b=2,c=10,d=5. Furthermore, we shall look in the following only at systems, which share the same common equilibrium, with the particular choice of $x_{0}+y_{0}+z_{0}=1$. This specific example has three eigenvalues of the Jacobian *J* given by $\lambda_{1}=0,\lambda_{2}=-9-2\sqrt{14},\lambda_{3}=-9+2\sqrt{14}$ with the corresponding right and left eigenvectors $v_{i}$ and ${\tilde{v}}_{i}$, respectively. The zero eigenvalue corresponds to a conserved quantity (implied by the condition x+y+z=1). The other two eigenvectors are negative, causing a dissipative nature of the dynamics and a stable equilibrium point, which, for this example, is given as $u^{e}={\left(2/5,1/5,2/5\right)}^{T}$.

**Figure 3:**
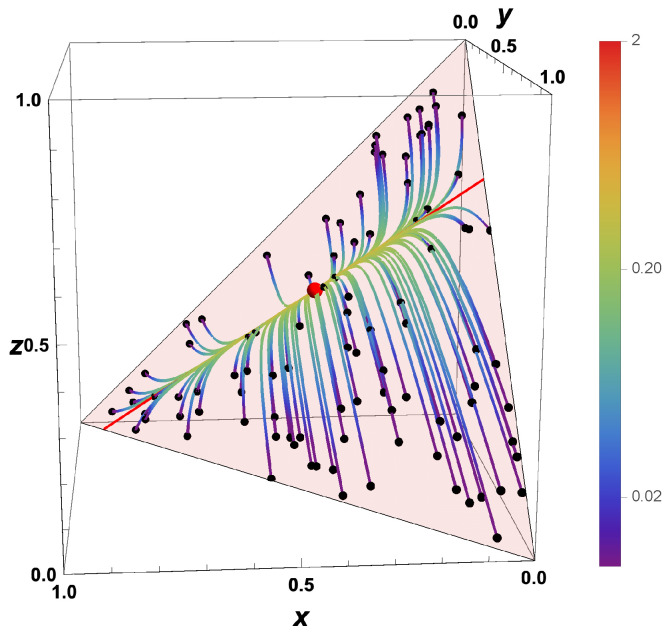
Thermokinetic trajectories for the simple linear example for t>0. Red line: intrinsic low-dimensional manifold. Shaded area: allowed subspace for the initial conditions x+y+z=1. Red point: Equilibrium value, Black points: Initial values, Color coding: time of reaction.

Now, let us look at the dynamic behavior of different systems starting at different initial values $u_{0}$. The behavior is shown by trajectories in the composition space (Figure 3) for randomly chosen (equidistributed) initial conditions.

It can be seen clearly that, after a first relaxation time (time is coded by the color), the states have evolved towards a lower-dimensional subspace (here a line), and after a second relaxation time towards the equilibrium point (which is attained for t→∞). This behavior for the simple example is reflected also in the behavior of general (nonlinear) reaction systems (cf. Figure 2). In this case, the analysis is more complex (because the Jacobian is not constant but depends on the state) but follows the same principle.

For the linear example shown here, the analysis is straightforward. The eigenvalue $\lambda_{1}=0$ governs the behavior $x+y+z=x_{0}+y_{0}+z_{0}$ (this represents the accessed space denoted by the red surfaces in the figures). Combining this condition with the condition that all chemical processes have relaxed

(17) $$\begin{array}{ccc} {\tilde{v}}_{1}u & = & {\tilde{v}}_{1}u_{0} \end{array}$$

(18) $$\begin{array}{ccc} {\tilde{v}}_{2}Ju & = & \lambda_{2}{\tilde{v}}_{2}u=0 \end{array}$$

(19) $$\begin{array}{ccc} {\tilde{v}}_{3}Ju & = & \lambda_{3}{\tilde{v}}_{3}u=0 \end{array}$$

yields the equilibrium point $u^{e}={\left(2/5,1/5,2/5\right)}^{T}$ (red dot in Figure 3), and assuming that only the fastest process (corresponding to $\lambda_{2}$) has relaxed

(20) $$\begin{array}{ccc} {\tilde{v}}_{1}u & = & {\tilde{v}}_{1}u_{0} \end{array}$$

(21) $$\begin{array}{ccc} {\tilde{v}}_{2}Ju & = & \lambda_{2}{\tilde{v}}_{2}u=0 \end{array}$$

we obtain the one-dimensional ILDM $u^{ILDM}\left(\theta \right)$ parameterized by the variable θ (red line in Figure 3, $u^{ILDM}\left(\theta \right)\approx {\left(\theta ,0.30\;-0.26\;\theta ,0.70\;-0.74\;\theta \right)}^{T}$).

### 2.3. The Behavior for Time Reversal

**Figure 4:**
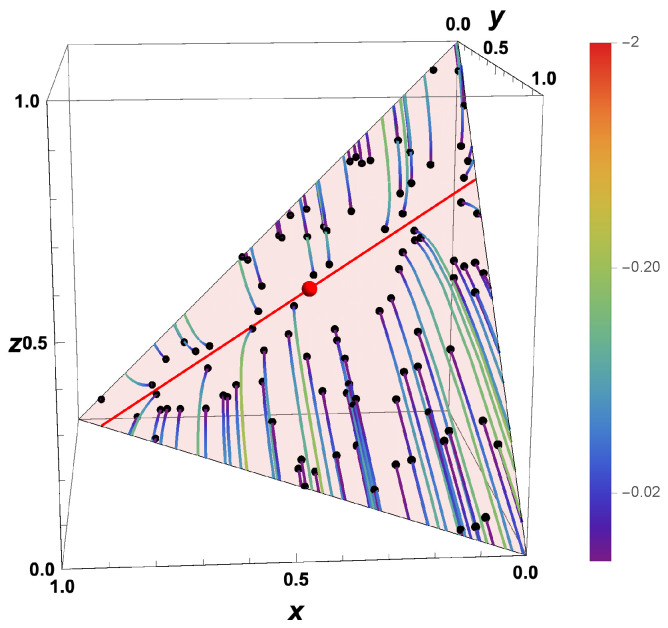
Thermokinetic trajectories for the simple linear example for t<0. Red line: intrinsic low-dimensional manifold. Shaded area: allowed subspace for the initial conditions x+y+z=1. Red point: Equilibrium value, Black points: Initial values, Colors: elapsed time.

For the moment, we assume that the time can be reversed for the macroscopic description of the chemical kinetics. If we know the thermokinetic state of a system at t=0, then can we determine the state that the system had at −Δt (with Δt>0) simply by integrating the system backwards in time. However, we have to know what happens for t→−∞. Is there a limiting value for t<0? Figure 4 shows the “backwards evolution” of states which are initially equi-distributed in the whole allowed domain. According to the governing equation system, there is no restriction of the accessed space. This means that states that initially belong to the allowed space can leave the allowed domain.

Note that, in this example, only trajectories up to $t=-\Delta t^{\partial \Sigma }$ are shown, where $\Delta t^{\partial \Sigma }$ denotes the reverse time after which the system attains a thermokinetic state on the boundary ∂Σ of the allowed domain. The fact that reverting the time causes the system according to the governing equation system to move out of the allowed domain (this is evident because Ω(ϕ∈∂Σ) points into the allowed domain, and consequently −Ω(ϕ∈∂Σ) points outwards) poses several questions:

- What happens for $t<-\Delta t^{\partial \Sigma }$?
- Does that mean that there is a “final time” for time going backwards (in contrast to time t→+∞ in forward direction?
- Why is this time different for different initial conditions?
- Why don’t the governing equations give any information on the “real behavior”?

**Figure 5:**
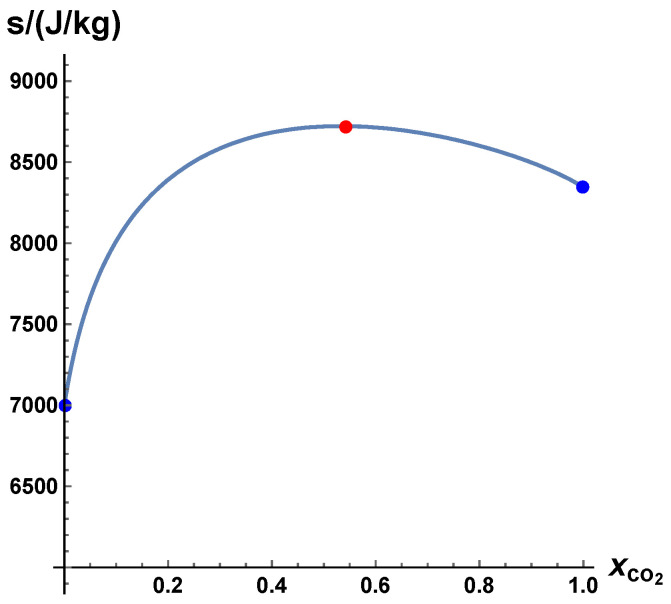
Dependence of specific entropy for an adiabatic isobaric system of ${\mathrm{CO}}_{2}$, CO, and $O_{2}$ with a molar C/O ratio of 1/2 and p= 1 bar, specific enthalpy h=−2512 kJ/kg (corresponding to a mixture of CO/$O_{2}$= 2/1 (molar) which has a temperature of 298 K). Red point: Equilibrium value.

We have to note, however, that this macroscopic kinetic behavior represents only a model for the real kinetics (which is based on microscopic processes, see below). On the other hand, a pure thermodynamic analysis shows the same behavior. Let us assume that we have a chemical system of ${\mathrm{CO}}_{2}$, CO, and $O_{2}$ (for simplicity of illustration, we omit the presence of oxygen atoms and ozone as species). If the molar composition is C/O is 1/2, then, for an adiabatic, isobaric system with p= 1 bar, T=−2512 kJ/kg (corresponding to a mixture of CO/$O_{2}$= 2/1 (molar), which has a temperature of 298 K), the dependence of specific entropy on the amount of ${\mathrm{CO}}_{2}$ is shown in Figure 5 (calculations were performed using the kinetics package HOMREA [50]). The maximum entropy (at the equilibrium point) is 8722 kJ/(K kg) (red symbol in Figure 5). For $x_{{\mathrm{CO}}_{2}}<x_{{\mathrm{CO}}_{2}}^{eq}$, the entropy is minimized for $x_{{\mathrm{CO}}_{2}}=0$, and, for $x_{{\mathrm{CO}}_{2}}>x_{{\mathrm{CO}}_{2}}^{eq}$, the entropy is minimized for $x_{{\mathrm{CO}}_{2}}=1$. Thus, if for time reversal entropy is decreasing in time, we end up at the boundaries of the domain.

However, how is the existence of intrinsic low-dimensional manifolds reflected in time reversal? A simple analysis of the Equation (15) under time reversal ($t\to t^{⋆}=-t$) yields

(22) $$\frac{du}{dt^{⋆}}=-Ju\;u\left(t=0\right)=u_{0}={\left(x_{0},y_{0},z_{0}\right)}^{T},$$

Replacing *J* by −J simply causes the eigenspaces to remain the same as in the “forward equation”, and the eigenvalues to change their signs. Zero eigenvalues (corresponding to conserved quantities) do not change, and eigenvalues <0 (corresponding to attractive processes) turn into eigenvalues >0 (repulsive processes). This is reflected in the plot of the evolution of the system backwards in time (Figure 4). The attracting low-dimensional manifold in the forward behavior turns into a separatrix, dividing the space into two areas, which evolve in the opposite direction towards the boundary ∂Σ. Furthermore, the fast repulsive modes lead to a movement in the direction of the fast subspace. Even for nonlinear governing equations, this fast subspace is typically linear [41,51].

## 3. Statistics of Macroscopic Systems

### 3.1. General Evolution Equation for the Probability Density Function

Let us now assume that the macroscopic thermokinetic states of the chemical reaction system are distributed initially according to a probability density function (this corresponds to having a large number *N* of macroscopic systems with composition vectors ${}^{⋆}\phi^{i}$, which do not interact (an interaction can be incorporated by adding appropriate mixing models [52,53]). Then, a transport equation for the probability density function $f\left(\psi ,t\right)d\psi =\mathrm{Prob}(\psi \leq {}^{⋆}\phi^{i}\left(t\right)<\psi +d\psi$), where < and ≤ are meant component wise for the vector, can be solved, which reads [53]

(23) $$\frac{\partial f(\psi ;t)}{\partial t}+\sum_{\alpha =1}^{n}\frac{\partial }{\partial \psi_{\alpha }}\left\{\Omega_{\alpha }f\left(\psi ;t\right)\right\}=0\;,$$

and the solution is obtained in a simple way by the method of characteristics (transformation from $\left(\psi ,t\right)\to \left(\psi^{0},t\right)$)

(24) $$\begin{array}{ccc} \frac{d\psi (\psi^{0},t)}{dt} & = & \Omega \left(\psi \left(\psi^{0},t\right)\right)\;\psi \left(t=0\right)=\psi^{0} \end{array}$$

(25) $$\begin{array}{ccc} \frac{df(\psi^{0},t)}{dt} & = & -f\left(\psi^{0},t\right)\sum_{\alpha =1}^{n}\frac{\partial \Omega_{\alpha }}{\partial \psi_{\alpha }}\left(\psi^{0},t\right)=-f\left(\psi^{0},t\right)\;\mathrm{trace}\left(\Omega_{\psi }\left(\psi^{0},t\right)\right)\;f\left(\psi^{0},0\right)=f^{0}, \end{array}$$

where $\Omega_{\psi }$ denotes the Jacobian of the chemical source term (${\left(\Omega_{\psi }\right)}_{ij}=\frac{\partial \Omega_{i}}{\partial \phi_{j}}$). Because $\mathrm{trace}(\Omega_{\psi })\leq 0$ for chemical reaction mechanisms is based on elementary reactions, any initial PDF thus relaxes to a Dirac δ-function $\delta (\psi -\phi^{e})$ located at the equilibrium point $\phi^{e}$.

### 3.2. The Consequences of Low-Dimensional Attracting Manifolds for the Evolution Equation for the Probability Density Function

The behavior for t→∞ reflects only part of the general observations made for the chemical kinetics. Thus, it is interesting to analyze how the other properties (e.g., relaxation to low-dimensional manifolds) are reflected in the statistics. If there is a large gap in the time scales of the slow and fast subspaces, it can be shown [19] that the chemical system relaxes very fast in the direction of the fast chemical subspace, while the slow processes are (to a good approximation) frozen. This results in a simplified evolution equation for the chemical system [42], and then we obtain for the evolution equation for the PDF (note that we drop the dependence $\left(\psi^{0},t\right)$ to simplify reading):

(26) $$\begin{array}{ccc} \frac{d\psi }{dt} & = & {\tilde{Z}}_{f}Z_{f}\Omega \end{array}$$

(27) $$\begin{array}{ccc} \frac{df}{dt} & = & -f\;\mathrm{trace}(Z_{f}\;{\tilde{Z}}_{f}\Omega_{\psi }), \end{array}$$

and, for t→∞, we obtain:

(28) $$\begin{array}{ccc} {\tilde{Z}}_{s}\psi & = & {\tilde{Z}}_{s}\psi^{0} \end{array}$$

(29) $$\begin{array}{ccc} 0 & = & {\tilde{Z}}_{f}\Omega \left(\psi \right) \end{array}$$

(30) $$\begin{array}{c} f\to \infty \end{array}$$

This means that, according to the fast chemical relaxation process, the PDF evolves such that f(ψ)→0 for $\psi \notin M^{m}$ and f(ψ)→∞ for $\psi \in M^{m}$.

### 3.3. Evolution Equation for the Probability Density Function of the Linear Test Case

For the simple example, the evolution of the PDF is readily obtained. Starting from (24), the evolution of the PDF yields

(31) $$\begin{array}{ccc} \frac{du}{dt} & = & Ju \end{array}$$

(32) $$\begin{array}{ccc} \frac{df}{dt} & = & -f\mathrm{trace}(J), \end{array}$$

and the evolution of the PDF according to the fast chemical process (slow processes frozen) yields:

(33) $$\begin{array}{ccc} \frac{du}{dt} & = & v_{2}{\tilde{v}}_{2}Ju=\lambda_{2}v_{2}{\tilde{v}}_{2}u \end{array}$$

(34) $$\begin{array}{ccc} \frac{df}{dt} & = & -f\lambda_{2} \end{array}$$

and for t→∞

(35) $$\begin{array}{ccc} {\tilde{v}}_{2}u & = & 0 \end{array}$$

(36) $$\begin{array}{ccc} {\tilde{v}}_{1}u & = & {\tilde{v}}_{1}u^{0} \end{array}$$

(37) $$\begin{array}{ccc} {\tilde{v}}_{3}u & = & {\tilde{v}}_{3}u^{0} \end{array}$$

(38) $$\begin{array}{ccc} f & \to & \infty \end{array}$$

This means (note that $\lambda_{2}<0$) that, according to fast chemical relaxation process, the PDF evolves such that f(ψ)→0 for ψ∉ILDM and f(ψ)→∞ for ψ∈ILDM. In the explicit formulation of the manifold equation, this allows for formulating *f* as

(39) $$f\left(\psi ;t\right)=\delta (\psi -\psi^{M}\left(\theta \right))g\left(\theta \right)$$

where δ denotes the Dirac delta function (used component wise for the vector elements), $\psi^{M}\left(\theta \right)$ the states on the manifold (parameterized by θ), and g(θ) a function describing the PDF along the manifold.

**Figure 6:**
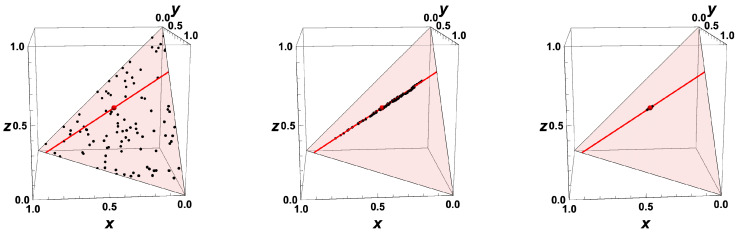
Thermokinetic states in composition space for the simple linear example at different times (left: t=0, middle: t=0.25, right: t=2), initial values distributed randomly in the allowed domain; Red line: intrinsic low-dimensional manifold. Shaded area: allowed subspace for the initial conditions x+y+z=1. Red point: Equilibrium value.

This behavior is reflected in the scatterplot for the kinetic systems (Figure 6). After a short time, the probability to find a state which is not close to the manifold tends to zero.

### 3.4. The Behavior for Time Reversal

**Figure 7:**
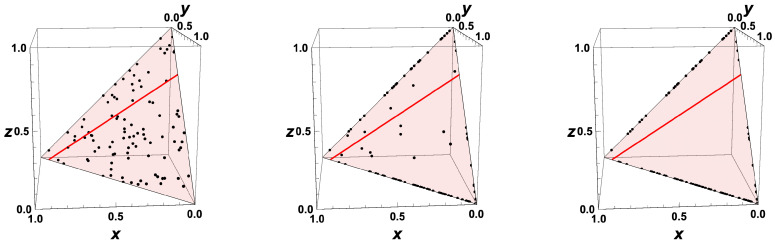
Thermokinetic states in composition space for the simple linear example at different times (left: t=0, middle: $t=-\min(0.1,\Delta t^{\partial \Sigma })$, right: $t=-\min(1,\Delta t^{\partial \Sigma })$, initial values distributed randomly in the allowed domain. Red line: intrinsic low-dimensional manifold. Shaded area: allowed subspace for the initial conditions x+y+z=1.

For macroscopic thermokinetic states distributed initially according to a probability density function, the behavior gets quite complicated for time going backwards. Starting from Equation (33), the evolution of the PDF after time reversal would lead to a PDF spread over the whole composition space with f→0. If we, however, account for the fact that the states are not allowed to leave the domain, we obtain a PDF given as f(ψ)→0 for ψ∉∂Σ and f(ψ)→∞ for ψ∈∂Σ. This behavior is shown in Figure 7 by a scatter plot of equi-distributed initial values. Furthermore, if time is reversed, then the fast processes correspond to a fast repulsion of initially close initial states. These fast processes are governed by the equation system

(40) $$\begin{array}{ccc} \frac{d\psi }{dt} & = & -{\tilde{Z}}_{f}Z_{f}\Omega \end{array}$$

(41) $$\begin{array}{ccc} {\tilde{Z}}_{s}\psi & = & {\tilde{Z}}_{s}\psi^{0}, \end{array}$$

which means that the processes in the slow subspace are frozen, and the evolution occurs only along the fast subspace. This fast subspace is typically close to linear for kinetic systems, which can be seen from the trajectories all moving approximately along the same direction (according to the direction of the fast eigenvector in this example, see Figure 4). The sign of the direction is determined by the low-dimensional manifold of the forward system. The low-dimensional manifold for the forward system turns into a separatrix for the backwards system.

**Figure 8:**
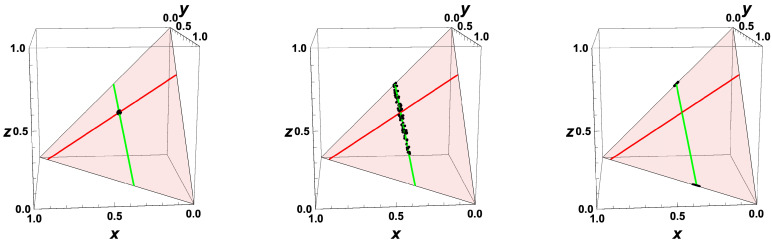
Thermokinetic states in composition space for the simple linear example at different times (left: t=0, middle: $t=-\min(0.2,\Delta t^{\partial \Sigma })$, right: $t=-\min(2,\Delta t^{\partial \Sigma })$), initial values distributed randomly in a circle of radius 0.014 around the equilibrium value; Red line: intrinsic low-dimensional manifold. Shaded area: allowed subspace for the initial conditions x+y+z=1. Red point: Equilibrium value, Green line: line through the equilibrium point in the direction of the eigenvalue largest in magnitude.

Now, it is interesting to investigate the behavior for system states which are initially close to the equilibrium value. This is illustrated in Figure 8, where the states for the example are shown for t=0, $t=-\min(0.2,\Delta t^{\partial \Sigma })$, and $t=-\min(2,\Delta t^{\partial \Sigma })$. If (like in Figure 8), the initial states are distributed in a very close neighborhood of the equilibrium point, then the states at subsequent times bunch along the line defined by the equilibrium point and the vector corresponding to the eigenvalue largest in magnitude (green line in Figure 8). For t→−∞, the final states bunch around two values on the boundary, and which final state is obtained is governed by the initial position relative to the separatrix.

These examples have shown that the statistics of the back relaxation get very complex and are inherently coupled with the questions raised above, namely: Is there a “final time” for time going backwards (in contrast to time t→+∞ in forward direction)? Why is this time different for different initial conditions? Why don’t the governing equations give any information on the “real behavior”?

Because the equations are deterministic and involve only forward causality, they do not allow for giving a full answer to these questions. The only information in this context that can be obtained from the governing equation system is the maximum time that a system had evolved in the past, and this maximum time is governed by the side condition that the system state is bound to the allowed space Σ. Note that, when the system is running backwards in time, species mass fraction becomes smaller than zero according to the governing equations. No physical information is embedded in the equation about rates for states not belonging to the allowed domain. This is a shortcoming of the macroscopic model.

## 4. The Microscopic View

In the sections above, we have assumed that there is a macroscopic formulation of chemical reactions, which is consistent with thermodynamics. It has been shown that, in principle (unless the system is in chemical equilibrium), we can get information on the past of the system if we know the present of the system. On the other hand, it has been shown that several paradoxical situations result. Therefore, it interesting to investigate whether these paradoxical situations arise from the fact that macroscopic rate equations are formulated, or whether they stem from peculiarities that are involved already in the microscopic formulation of chemical kinetics.

In the macroscopic description of chemical kinetics, only rate equations for the species concentrations have to be solved. However, these kinetic equations rely on thermal equilibrium. This means that, on a microscopic level, vibrational, rotational, and translational degrees of freedom are in thermal equilibrium, governed by a Boltzmann distribution for translational, rotational, and vibrational degrees of freedom of the molecules considered, defining one common temperature. The reason that this assumption is a good approximation is that typically the relaxation to thermal equilibrium occurs with rates much faster than the chemical reaction itself. If this assumption is no longer valid (typically the vibrational relaxation is the slowest process of thermalization), state selective reaction mechanisms have to be used for the description of the system [9,10,11].

In principle, the kinetics of single elementary reactions are based on an interaction of different molecules in different microscopic states (translational energies, rotational, vibrational, and electronic states). Rates of bimolecular reactions can typically be obtained based on collision or transition state theory [7,54]. In its simplest formulation, the theory states that the reaction rate is determined both by the collision frequency and by the fraction of those collisions that lead to successful reaction. Both necessary conditions (collision and reaction) are typically determined assuming that the energy distribution of the different species can be described by a Boltzmann-distribution. This means that the macroscopic rate constant can be readily obtained, when a local thermal equilibrium can be assumed. On the other hand, any reactions involving a non-thermal distribution have to be described by more complex models based on the solution of master equations. In the case of uni-molecular reactions or multiple-well reactions, energy transfer between collisions requires the solution of a chemical master equation [6,7,55]. In the following, we shall focus only on models based on the solution of the master equation because these represent typical examples.

### 4.1. Unimolecular Reactions—Master Equation

There are a variety of different formulations of the master equation [7], and, although, in many cases, the master equation is cast in a continuum form, we shall investigate the discrete form because it allows an analysis similar to the previous sections. We adopt a simple formulation according to [56], which is based on a discretization into energy parcels. Then, we obtain a finite dimensional ordinary differential equation system for the vector $n={\left(n_{1},n_{2},\ldots ,n_{n_{E}}\right)}^{T}$ of average number densities in energy interval *i* out of a total of $n_{E}$ energy intervals [56].

(42) $$n_{i}=\frac{1}{E_{i+1}-E_{i}}\int_{E_{i}}^{E_{i+1}}n\left(E\right)dE$$

The rate of change of each energy parcel is given by

(43) $$\frac{dn_{i}}{dt}=\sum_{i}\left(p_{i,j}n_{j}-p_{j,i}n_{i}\right)$$

or in vector notation

(44) $$\frac{dn}{dt}=R_{a}f+\left(\omega \left(P-I\right)-K\right)n=R_{a}f+Jn$$

where $R_{a}$ denotes the rate of reaction that produces the species (input flux), *f* is the corresponding distribution, $P_{i,j}$ the transition probability form energy interval *j* to energy interval *i*, I the identity matrix, and *K* the diagonal matrix of averaged unimolecular rate coefficients $K_{ii}$, and ω denotes the collision frequency with bath gas molecules. If $R_{a}f=0$ (no reaction producing the species) and K=0 (no reaction of the species to other molecules), this equation describes the relaxation of the energy distribution towards a Boltzmann-distribution. In this case, one eigenvalue of the Jacobian is 0 (corresponding to species conservation), while all other eigenvalues are negative [57]. There are several important properties of the matrix ω(P−I), which are discussed, e.g., in [57]). If K≠0 (chemical reaction of the species to products), then the eigenvalues shift, but they are still negative (can be verified using the arguments used in [57] for determining the Gershgorin circles). Even in the case of chemical reaction, the eigenvalues are typically well separated from the largest one (the one governing the “macroscopic” behavior), unless there are very special conditions such as very high temperatures [56].

If we assume that the overall Jacobian *J* is regular (note that the chemical equation to products removes the species conservation condition), and $R_{a}f$ is constant, the solution is given by:

(45) $$n=e^{Jt}\left(n^{0}+J^{-1}R_{a}f\right)-J^{-1}R_{a}f,$$

which can easily be verified by insertion. We see that this equation resembles very much the equations governing the macroscopic behavior, although there are several differences: If there is no influx term for the species’ energy parcels ($R_{a}f=0$), all number densities tend to 0 for t→∞. If there is an influx, then a non-autonomous ordinary differential equation system results. In this case, the Jacobian differs from the global quasi linearization matrix. However, if there still exists a global steady state, the analysis can still be applied. Therefore, we can easily transfer results from the macroscopic behavior to the microscopic behavior.

Nevertheless, we have to note that there are important properties of this equation system and its solution:

- it is an initial value problem
- it is an $n_{E}$-dimensional system of stiff ordinary differential equations with all eigenvalues of the Jacobian being negative
- The “rates” $P_{ij}$ for the pure non-reacting system are related to each other using the law of detailed balance, i.e., they assume that the equilibrium obeys a Boltzmann-distribution [57]
- the system is “deterministic”

If we look at a typical spectrum of the eigenvalues (e.g., for ethane dissociation [58]), it can be observed that many eigenvalues are large in magnitude compared to the smallest non-zero. Comparing with the macroscopic rate equation, we see that the qualitative behavior is very similar: The system dynamics’ approaches lower- and lower dimensional manifolds and finally (in the case of a chemical reaction ($K_{ii}\neq 0$) leads to a complete formation of the products). This means that we can transfer all the knowledge on the qualitative behavior from the macroscopic case to the microscopic case.

### 4.2. The Behavior for Time Reversal

A straightforward analysis shows that the Jacobian of the deterministic system for time going backwards ($t^{*}=-t,t<0$) turns to $J^{*}=-J$ and the solution is given by:

(46) $$n=e^{-Jt^{*}}\left(n^{0}-J^{-1}R_{a}f\right)+J^{-1}R_{a}f.$$

Replacing *J* by −J simply causes the eigenspaces to remain the same as in the “forward equation”, and the eigenvalues to change their signs. Zero eigenvalues (corresponding to conserved quantities) do not change, and eigenvalues <0 (corresponding to attractive processes) turn into eigenvalues >0 (repulsive processes), cf. the plot of the evolution of the system backwards in time (cf. Figure 4 for the macroscopic case). The attracting low-dimensional manifold in the forward behavior turns into a separatrix, dividing the space into two areas, which evolve in opposite directions towards the boundary ∂Σ. For system states which are initially close to the equilibrium value, the states at subsequent times bunch along the line defined by the equilibrium point and the vector corresponding to the eigenvalue largest in magnitude (cf. Figure 7). For $t^{*}\to \infty$ (t→−∞), the final states bunch along the boundary, and which final state is obtained is governed by the initial position relative to the separatrix.

## 5. The “Submicroscopic” Level

The microscopic level is based on an analysis of the dynamics of different translational, rotational, vibrational, and electronic states of the atoms and molecules. These states are typically obtained by quantum mechanics calculations [7,55]. In these calculations for potential energy surfaces to obtain transition state, there are, however, also steady state approximations involved, such as the Born–Oppenheimer (electronically adiabatic) approximation, equivalent to assuming that the motion of the atoms does not cause real or virtual transitions between different electronic states [7]. This further complicates the analysis, and shall not be discussed here.

## 6. Bridging the Gap between Microscopic and Macroscopic Behavior

The splitting of the different hierarchical level (systems, macroscopic, microscopic) is based on the observation that there exists a disparity of time scales (note that we do not discuss here the “submicroscopic” level). Most of the time-scales are dissipative in nature (corresponding to negative eigenvalues of the Jacobians). This causes a relaxation of the chemical dynamics towards lower- and lower dimensional attracting manifolds until, for t→∞, the equilibrium is attained. The processes occurring on the microscopic level are typically very fast, and it can be assumed (to a very good approximation) that only the slowest time scale (largest eigenvalue) governs the overall elementary reaction (see above). Therefore, a splitting is possible from the microscopic to the macroscopic level. However, what happens for a time reversal?

### 6.1. Microscopic Processes on a Macroscopic Level

In principle, it is possible to write down a set of master equations for all the states of all the different molecules. This, however, leads to an enormous size of the resulting differential equation systems. Nevertheless, we can write formally such a combined system and analyze the consequences for the dynamics. Let us assume that there are $n_{s}$ different chemical species α, where each of the chemical species is again “discretized” into $n^{\alpha }={\left(n_{1}^{\alpha },n_{2}^{\alpha },\ldots ,n_{n_{E}^{\alpha }}^{\alpha }\right)}^{T}$ of average number densities in energy interval *i* out of a total of $n_{E}^{\alpha }$ energy intervals. For each chemical species α, we obtain rate equations according to (note that we neglect here species influx other than that resulting from production from other species):

(47) $$\frac{dn^{\alpha }}{dt}=\left\{\omega \left(P^{\alpha }-I\right)-\sum_{\beta \neq \alpha }K^{\alpha \beta }\right\}n^{\alpha }+\sum_{\beta \neq \alpha }K^{\beta \alpha }n_{\beta }$$

where $P^{\alpha }$ is the transition matrix for species α, I the identity matrix, and $K^{\alpha \beta }$ the matrix of unimolecular rate coefficients, where $K_{ij}^{\alpha \beta }$ denotes the energy-specific rate constant of the *i*-th state of molecule α yielding the *j*-th state of molecule β.

This yields a Jacobian for the whole system, which is given by

(48) $$J^{\alpha \alpha }=\left\{\omega \left(P^{\alpha }-I\right)-\sum_{\beta \neq \alpha }K^{\alpha \beta }\right\}\;\;J^{\alpha \beta }=K^{\beta \alpha }.\;\;\mathrm{for}\;\;\beta \neq \alpha$$

(49) $$J=\left(\begin{array}{cccc} J^{11} & J^{12} & \cdots & J^{1n_{s}}\\ J^{21} & J^{22} & \cdots & J^{2n_{s}}\\ \vdots & \vdots & \ddots & \vdots \\ J^{n_{s}1} & J^{n_{s}2} & \cdots & J^{n_{s}n_{s}} \end{array}\right)$$

where each block $J^{\alpha \beta }$ has dimension $n_{E}^{\alpha }\times n_{E}^{\beta }$. A detailed analysis of the Jacobian is a challenging task. Therefore, we shall try to use physical arguments for the qualitative analysis. First of all, we note that the diagonal blocks of *J* in Equation (49) govern the fast relaxation processes of the energy parcels and the slow reaction processes. On the other hand, the off-diagonal blocks of *J* describe the slow reactions among different species. Therefore, we expect (without providing any proof) that at least the spectrum of the fast processes does not change very much. Noting that the reactions act only as a small perturbation *J* is block diagonally dominant and can be represented by a block diagonal matrix plus a full perturbation matrix. Because of the eigenvalue gap between the slow and the fast subspace, the fast subsystem will change only a little. It will still govern the energy transitions, and the slow subspace will govern the reactions among species.

### 6.2. The Behavior for Time Reversal

Again, a formal time reversal causes the eigenvalues of the Jacobian of the system to change their sign. The repulsive fast subspace is then governed by the energy transfer reactions, whereas the slow subspace (in general nonlinear) is governed by the slow reaction processes. If we compare the behavior with time reversal in the macroscopic case, we see that the fast energy transfer reaction leads to a fast evolution towards the boundary ∂Σ of the allowed domain. Because the energy transfer processes correspond to the eigenvalues largest in magnitude, the boundary will first be reached because certain energy parcels attain zero number densities and not because species concentrations become zero. This means that the maximum negative time will be governed by the very fast processes on the microscopic level.

## 7. Discussion and Conclusions

The analysis presented above has shown that there exists a hierarchy for the chemical kinetics not only of the different levels (from the system level down to the microscopic level), but also within the different levels. The whole chemical kinetics are characterized by a disparity of time scales. In typical applications, the different hierarchical levels thus can be decoupled from each other by a consequent use of time scale splitting. For the forward behavior, all hierarchical levels considered have in common the following features:

- They are governed by large systems of stiff nonlinear ordinary differential equations
- The governing equations constitute an initial value problem
- The solutions are unique for given initial values
- The system is “deterministic”
- The trajectories do not cross in the thermokinetic space
- The trajectories evolve in time towards an equilibrium
- The disparity of time scales allows a decoupling of slow and fast processes
- Most time scales have a dissipative nature
- There is a transient relaxation towards low-dimensional manifolds

For a simple time reversal, due to the deterministic nature of the equations, the behavior backwards in time shows the following features for all hierarchical levels:

- Due to the deterministic nature of the kinetic equations, the question “What was the state of a system at a time before?” can be answered based on the knowledge of the state at a current time.
- The trajectories evolve in time towards the boundary of the allowed domain.
- No information is contained in the equation system for $t<-\Delta t^{\partial \Sigma }$, where $t=-\Delta t^{\partial \Sigma }$ is the time when states reach the boundary of the allowed domain.
- Eigenvalues <0 of the Jacobians or the GQL matrix) (corresponding to attractive processes) turn into eigenvalues >0 (repulsive processes).
- Most eigenvalues of the backwards system are positive, corresponding to repulsive processes.
- The disparity of time scales allows a decoupling of slow and fast processes.
- An attracting low-dimensional manifold for the forward system turns into a separatrix for the backwards system.
- For t→−∞, the final states bunch along the boundary, and which final state is obtained is governed by the initial position relative to the separatrix.

Note, however, that the systems analyzed are based on a deterministic description of the chemical kinetics on all hierarchical levels investigated. Furthermore, all the kinetic models involve assumptions about the direction of time (e.g., assumptions on the relaxation of energy distributions towards a Boltzmann-distribution). Thus, it is clear that the analysis can address questions like “What was in the past?” (deterministic view back in time), but only given the forward system is deterministic (causality of reactions [12]).

## Abbreviations

The following abbreviations are used in this manuscript:

PDF	probability density function
ILDM	intrinsic low-dimensional manifolds
GQL	global quasi-linearization

## Appendix A. Eigen System and Solution for the Simple Linear Test Case

(A1) $$\begin{array}{cccc} \frac{dx}{dt} & = & -ax+by & \\ \frac{dy}{dt} & = & ax+by & -cy+dz\\ \frac{dz}{dt} & = & \; & cy-dz, \end{array}$$

With $u={\left(x,y,z\right)}^{T}$, the equation reads:

(A2) $$\frac{du}{dt}=\underset{J}{\underset{︸}{\left(\begin{array}{ccc} -a & b & 0\\ a & -b-c & d\\ 0 & c & -d \end{array}\right)}}u=Ju\;u\left(t=0\right)=u_{0}={\left(x_{0},y_{0},z_{0}\right)}^{T},$$

The analytical solution is given by:

(A3) $$u=e^{Jt}u_{0}=Ve^{\Lambda t}V^{-1}u_{0}$$

For the case of a=1,b=2,c=10,d=5, the matrices are given as:

(A4) $$\begin{array}{ccc} V & = & \left(\begin{array}{ccc} 1 & \frac{1}{5}\left(\sqrt{14}-3\right) & \frac{1}{5}\left(-3-\sqrt{14}\right)\\ \frac{1}{2} & \frac{1}{5}\left(-2-\sqrt{14}\right) & \frac{1}{5}\left(\sqrt{14}-2\right)\\ 1 & 1 & 1 \end{array}\right) \end{array}$$

(A5) $$\begin{array}{ccc} \Lambda & = & \left(\begin{array}{ccc} 0 & 0 & 0\\ 0 & -9-2\sqrt{14} & 0\\ 0 & 0 & 2\sqrt{14}-9 \end{array}\right) \end{array}$$

(A6) $$\begin{array}{ccc} V^{-1} & = & \left(\begin{array}{ccc} \frac{2}{5} & \frac{2}{5} & \frac{2}{5}\\ \frac{9}{10\sqrt{14}}-\frac{1}{5} & \frac{1}{35}\left(-7-4\sqrt{14}\right) & \frac{1}{140}\left(42-\sqrt{14}\right)\\ -\frac{1}{5}-\frac{9}{10\sqrt{14}} & \frac{1}{35}\left(4\sqrt{14}-7\right) & \frac{1}{140}\left(42+\sqrt{14}\right) \end{array}\right) \end{array}$$

(A7) $$\begin{array}{ccc} e^{\Lambda t} & = & \left(\begin{array}{ccc} 1 & 0 & 0\\ 0 & e^{-\left(9+2\sqrt{14}\right)t} & 0\\ 0 & 0 & e^{\left(2\sqrt{14}-9\right)t} \end{array}\right) \end{array}$$
